# Editorial

**Published:** 1856-06

**Authors:** 


### THE MEDICAL EXAMINER.

### PHILADELPHIA, JUNE, 1856.

A very large portion of our space in this number is devoted to the publication of the proceedings of the last meeting of the American Medical Association. In its preparation, not having had the pleasure to be present ourselves, we have mainly adopted the excellent Report published in the "Detroit Free Press," which we are informed may be generally relied on, both in regard to its fulness and accuracy. We take great pleasure, also, in laying before our readers the very able and eloquent address of the retiring President, Dr. Wood; an address which we are confident will be received by the profession everywhere, as it was listened to in Detroit, with unmingled gratification.

The number of delegates present at the meeting, we learn, was about three hundred, the largest portion of whom were from the West and North-West. The proceedings, we are happy to state, were exceedingly harmonious, and the duties of the Chair, upon which so much depends, were discharged by the President elect, Dr. Pitcher, with dignity and courtesy. The Reports were in many instances able and interesting. None were listened to with more attention than the two from the Committees on Medical Literature. The first presented was by Dr. Gross, of Kentucky, who was appointed at the Session of 1855. The Report urged upon the schools the duty of encouraging American Authors by recommending their works, as text-books for their pupils, and strongly condemned the practice of editing English works, and putting the Editor's name to the reprint. The author of the report did not wish to discourage the use of foreign works, but thought they should be employed, principally, as books of reference, not as text-books.

The report presented on the same subject by Dr. Breckenridge, also of Kentucky, took an opposite view, and recommended "Free Trade" in every way.

Two important amendments to the Constitution were proposed by one of the delegates from Philadelphia, both of which, or somewhat similar changes, strike us as desirable modifications of the present system. One of these proposes that the Association shall still meet annually, and while it still maintains its migratory character, shall have some of the advantages of permanency, by meeting once in two or three years in some fixed place, say, Washington, Philadelphia or New York, where its archives shall be kept, and not be, as is now the case, scattered over the various cities of the Union, in which the Association has already held or may hereafter hold its meetings.

The other amendment suggests one permanent Secretary, and two assistants, one of the latter to be chosen, as at present, from the city where the Association is about to meet. With a view of securing the attendance of the permanent Secretary at all meetings, it is also proposed to pay his expenses to and from the place of meeting, and while attending the same. The advantages of such an officer are too obvious in all well-regulated societies, to need any enumeration.

The afternoon session of Wednesday was, we understand, dispensed with to accept the invitation of the Committee of Arrangements, who hospitably entertained the Association on board of the Steamer Western World. This truly magnificent steamer was filled with the beauty and fashion of Detroit. The sail up Lake St. Clair and back into Lake Erie, we learn, was most delightful, and marred only by the rain which fell unceasingly during the whole period. The entertainment provided was on a most generous scale and no one who was present can fail to remember with pleasure the afternoon spent in the excursion on the Lake.

The Association were handsomely entertained on Tuesday evening at the houses of Drs. Morse Stuart and H. S. Cobb, and of Messrs. Edmund A. Brush and Albert Crane; and on Thursday evening by Dr. Z. Pitcher, and Messrs. Chas. Howard and H. Ledyard. At the latter place, an invitation was extended to the delegates to proceed to the residence of Mrs. Canfield, where an opportunity was afforded them of viewing some exquisite paintings and statuary.

The weather, during part of the time, was most unpropitious. An easterly wind and drizzling rain prevailing during Tuesday, which latter increased on Wednesday till it poured in torrents.

Although too early in the season to see Detroit to advantage, the impression left upon the minds of all who visited it was, that it is a beautiful city, its society very agreeable, and characterised by the courtesy, kindness, and true hospitality of its inhabitants.

